# Spectral Characteristics of EEG during Active Emotional Musical Performance

**DOI:** 10.3390/s21227466

**Published:** 2021-11-10

**Authors:** Jachin Edward Pousson, Aleksandras Voicikas, Valdis Bernhofs, Evaldas Pipinis, Lana Burmistrova, Yuan-Pin Lin, Inga Griškova-Bulanova

**Affiliations:** 1 Jāzeps Vītols Latvian Academy of Music, LV-1050 Riga, Latvia; jachin.edward.pousson@jvlma.lv (J.E.P.); valdis.bernhofs@jvlma.lv (V.B.); lana.burmistrova@jvlma.lv (L.B.); 2 Department of Neurobiology and Biophysics, Vilnius University, LT-10257 Vilnius, Lithuania; aleksandras.voicikas@gmc.vu.lt (A.V.); evaldas.pipinis@gmc.vu.lt (E.P.); 3 Institute of Medical Science and Technology, National Sun Yat-sen University, Kaohsiung 80424, Taiwan; yplin@mail.nsysu.edu.tw; 4 Department of Electrical Engineering, National Sun Yat-sen University, Lienhai Road, Kaohsiung 80424, Taiwan

**Keywords:** emotion, active performance, EEG

## Abstract

The research on neural correlates of intentional emotion communication by the music performer is still limited. In this study, we attempted to evaluate EEG patterns recorded from musicians who were instructed to perform a simple piano score while manipulating their manner of play to express specific contrasting emotions and self-rate the emotion they reflected on the scales of arousal and valence. In the emotional playing task, participants were instructed to improvise variations in a manner by which the targeted emotion is communicated. In contrast, in the neutral playing task, participants were asked to play the same piece precisely as written to obtain data for control over general patterns of motor and sensory activation during playing. The spectral analysis of the signal was applied as an initial step to be able to connect findings to the wider field of music-emotion research. The experimental contrast of emotional playing vs. neutral playing was employed to probe brain activity patterns differentially involved in distinct emotional states. The tasks of emotional and neutral playing differed considerably with respect to the state of intended-to-transfer emotion arousal and valence levels. The EEG activity differences were observed between distressed/excited and neutral/depressed/relaxed playing.

## 1. Introduction

Music is known to have a capacity to impact mood, and it is ubiquitously used for this purpose as an affective medium. However, the perception, aesthetic assessment, and induction of specific emotions from a musical signal constitute a complex individual process [1,2]. For example, Juslin and Västfjäll (2008) [3] proposed six physiological mechanisms of emotion induction by music based on the existing body of literature, including brain stem reflexes, evaluative conditioning, emotional contagion, visual imagery, episodic memory, and musical expectancy. The failure to control for these underlying mechanisms may lead to inconsistent or non-interpretable findings [4,5]. Indeed, the proposed mechanisms of emotion induction by music differ regarding many aspects including the information focus, cultural impact, dependence on musical structure, etc. These are important from the listener’s perspective. However, these are also implicated in intended emotion transfer while performing.

The majority of research in this area focuses on the assessment of emotions evoked by music. However, the research on neural correlates of intentional music transfer by the performer is still limited [6,7]. In music, violations of expectation (surprises) are often a critical feature of its emotional content and is tightly related to creativity and improvisation. A network of prefrontal brain regions commonly linked to improvisatory behavior in music includes the presupplementary motor area, medial prefrontal cortex, inferior frontal gyrus, dorsolateral prefrontal cortex, and dorsal premotor cortex [8]. While the systematic variations in acoustic parameters that differentiate one expressive performance from another have been investigated in detail (e.g., Clarke, 1988) [9], the neurobiological underlying mechanisms of emotional communication are not that well investigated. In part, this is due to methodological limitations imposed by currently available neuroimaging methods. In the initial fMRI study, McPherson et al. (2016) [10] found that the creative expression of emotions through music engages emotion-processing areas of the brain in ways that differ from the perception of emotion in music. However, electroencephalogram (EEG), representing a unique real time brain activity assessment method, would be more beneficial in this context and allow for more ecologically valid settings.

In this study, we attempted to evaluate EEG patterns recorded from musicians who were instructed to perform a simple piano score while manipulating their manner of play to express specific contrasting emotions and self-rate their communication of the target emotion on the scales of valence (describes the extent to which an emotion is positive or negative) and arousal (the physiological alertness associated with the associated emotional state) [11]. In embodied music performance contexts, the emotion intended to be communicated by musicians does not necessarily reflect their actual felt emotions [12,13,14]. Thus, subjects were requested to rate their own performance not according to how they actually felt while playing but how well they felt they imbued their performance with the intended emotion.

Despite growing number of attempts to classify emotions from EEG [15,16,17], there is no standard methodology in the current body of work related to music, both in respect to the experimental setup and evaluation of EEG signal parameters. The existing reports are somewhat difficult to interpret, reproduce, or relate to known physiological processes. Moreover, only few studies related to music improvisation or active playing [18,19,20]. Thus, we also aimed to control several methodological aspects that potentially should lead to better interpretation of findings. First, the theme provided to the subjects was designed to be as neutral and universal as possible; an extended pentatonic scale was used to avoid the implicit tendency in Western classical functional harmony to gravitate towards a tonic through subdominant and dominant tensions as a means to convey intent [21], thereby finding more common ground with musical systems used across different cultures historically and today [22,23]. The piano score used in the study was also designed to take into consideration that music improvisation in real-life requires an establishment of a theme to variate upon, and thus subjects were asked to perform the music as written before variation with expressive cues. Second, we contrasted emotional playing to neutral playing to account for movements occurring during performance and minimize their effect on EEG emotion-related correlates and to provide an ecologically valid context such as in a music studio, or concert hall [24]. The experiment took place in a common practice room within the music academy modified for the EEG recording procedure. Audio of all performances were recorded, and subjects were made aware these were to be evaluated by listeners at a later date. All of the above-mentioned steps were taken to ensure the musical performance indeed embodied within an ecologically valid context. We applied spectral analysis to the signal as an initial step to be able to connect findings to the wider field of music-emotion research.

## 2. Materials and Methods

### 2.1. Participants

Ten healthy subjects (2 males and 8 females; age 19–40 years.) were recruited to participate in a piano-playing task over four sessions taking place on different days. All subjects were experienced piano players with a minimum of five years of academic training. Four of the subjects specialized in piano studies while the other six specialized in other instruments and fields, but played the piano regularly as part of their profession or ongoing training. The study was approved by Rīga Stradiņš University Research Ethics Committee (Nr.6-1/01/59), and all participants gave their written consent.

### 2.2. Experimental Design and Procedure

**Figure 1:**
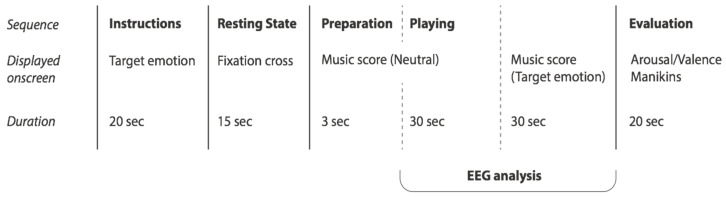
The schematic representation of the experimental trial.

**Figure 2:**
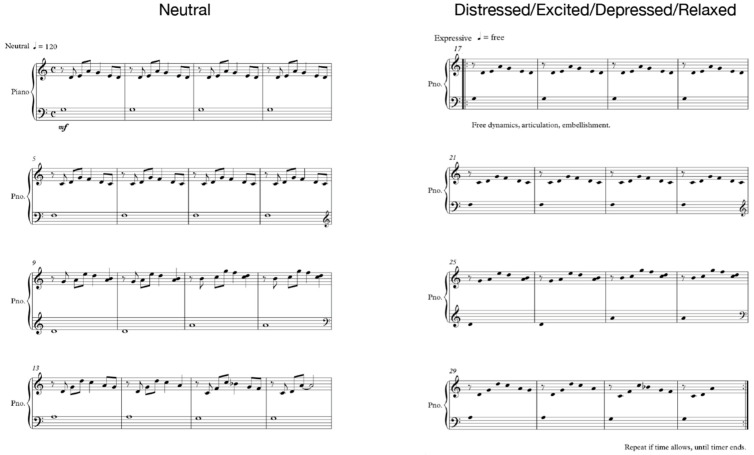
Piano scores used in the study.

The experiment was controlled with the Psychopy stimulus presentation software [25]. The scheme of a single experiment trial is plotted in Figure 1. Within a single session, each participant was instructed to play the same musical score (self-composed by the author, Figure 2) while intentionally expressing one of five emotional states (excited, distressed, depressed, relaxed, neutral) based on a 2D valence-arousal emotion model [11]. The musical score was designed to be simple enough for an experienced player to quickly grasp and create variations upon, and was arranged in two pages. The first page was to be played neutrally or mechanically, in tempo, lasting 30 s (further used as a baseline). The second page was a repeat of the first but with freedom to use tempo, rhythm, articulation, embellishment and any other expressive cues at the player’s disposal to express the target emotion through their performance. The sequence of emotional states was randomly ordered for each trial.

The subjects were briefed before their first session regarding the sequence of the recording protocol, the piano playing task, the chosen emotional descriptors, and self-evaluation step. The words adapted to describe each quadrant of the 2D model of affect were in English, and were chosen with consideration for how they may be interpreted by an international group of participants. The researchers verified at each participant’s first session that the task and descriptors were fully understood by conducting a test run with time to ask questions and adjust for signal quality. The detailed description of the instruction procedure is presented in the Supplementary Material.

**Figure 3:**
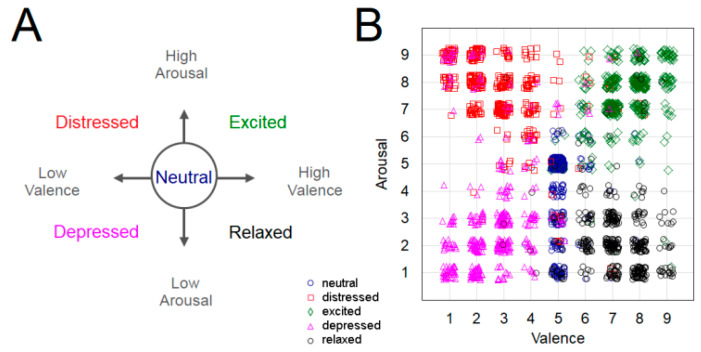
(**A**) Emotion descriptors used for performance instructions; low valence scores (<5) correspond to negative and high scores (>5) to positive valence. (**B**) Results of subjective self-assessment on the experienced emotional arousal and valence levels for each experimental trial of every participant.

During the experiment, participants were instructed to remain seated and fully attend to their expressive intentions. Subjects were first presented the target emotion for 20 s onscreen (Figure 3A). This was followed by a resting state recording for 15 s with a fixation cross displayed onscreen. Following the resting state period, the music score appeared onscreen and subjects were given 3 s to place their hands on the piano keyboard before beginning to play. Subjects played the score through neutrally (further used as a baseline) for 30 s followed by 30 s of expressive playing as instructed at the beginning of the run. Following the piano-playing period, subjects were required to rate their own performance on a nine-point scale (1–9) of emotional valence (from negative/low to positive/high) and emotional arousal (from low to high) with neutral represented on both scales as 5. Subjects were asked not to rate their actual felt emotions but rather how they felt their performance reflected the intended target emotion. When errors were occasionally made by participants at the self-rating step, the researchers were made aware and corrected these entries in the collected data manually.

While recording, trials were repeated five times in a row, making up one run. Subjects were given a brief rest between each run, also allowing time for any necessary adjustments for maintaining EEG signal quality. Five runs (total of 50 trials) were recorded on each of the four sessions attended by each subject. For the analysis of EEG correlates with emotional state, only piano-playing excerpts (30 s neutral baseline plus 30 s emotional) with self-reported valence and arousal labels followed by each piano-playing trial were used. Subjects were informed that the audio recordings of their performance were to be evaluated by listeners at a later date.

### 2.3. EEG Acquisition

A 32-channel Enobio 32 system was used to recorded EEG signals. Electrodes were placed according to the International 10–20 system, with Common Mode Sense (CMS) and Driven Right Leg (DRL) connections applied to the right earlobe for electrical grounding. Signal quality was monitored via a quality index consisting of four parameters; line noise, main noise, offset and drift; provided within Enobio’s native signal acquisition software Neuroelectrics Instrument Controller v.2.0.11.1 (NIC). EEG data was recorded at a 500 Hz sampling rate with a 50 Hz notch filter applied to remove power line noise.

### 2.4. EEG Preprocessing

The off-line data pre-processing was performed with custom written scripts implementing functions available in MNE-Python [26] for cleaning. Raw EEG data were filtered with band-pass filter (FIR, 1–45 Hz) and re-referenced to the average reference. Independent component analysis was used to correct eye-movements. Data was segmented into 2 s epochs with 50% overlap starting from 5 to 25 s relative to the start of the playing trial. Further data cleaning was performed using a fully automated approach implemented in the autoreject package (version 0.1) using default settings [27]. Identified bad sensors and periods containing artifacts were discarded, resulting in 10% of data removed. The removed EEG channels were reconstructed using spherical spline interpolation [28].

### 2.5. EEG Analysis

The further data pre-processing and analysis was performed with custom written scripts implementing functions available in Fieldtrip [29], following major steps as described in [30]. For each individual and condition average power spectra were computed based on fast Fourier transformation (FFT) of the segmented data after the application of a Hanning taper [30]. Power was calculated in delta (1–4 Hz), theta (4–8 Hz), alpha (8–12 Hz), beta (12–30 Hz) and gamma (30–45 Hz) frequency bands. The relative power was derived as a measure of interest by dividing the average power during emotional play by the average power of the preceding baseline neutral play. All measures were combined across four sessions. To test for statistical differences between the emotional conditions, non-parametric permutation tests with a cluster-based correction for multiple comparisons were employed (5000 permutations, *p* < 0.05, two sided) on all channels [31].

## 3. Results

The results of subjective evaluation on the scales of valence and arousal are plotted in Figure 3B.

**Figure 4:**
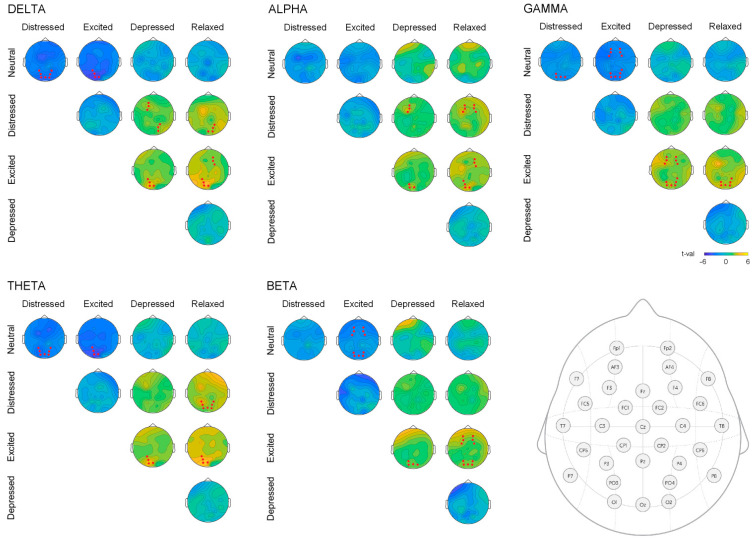
Topographical plots of T values for different emotional conditions (neutral, distressed, excited, depressed and relaxed) and for EEG bands (delta (1–4 Hz), theta (4–8 Hz), alpha (8–12 Hz), beta (12–30 Hz), and gamma (30–45 Hz). Red dots represent electrode clusters where differences between conditions were significant. For convenience, electrode locations are plotted on the bottom right.

The topographical plots of T values when contrasting playing conditions separately for each frequency band are presented in Figure 4. The electrode clusters where significant differences were obtained are marked in red. Further the detected differences are reported presenting mean values of the cluster and standard deviation measures.

Excited and distressed conditions did not differ in any EEG measures. However, significant differences were observed in comparison to all other playing instructions. Reduced levels of parieto-occipital delta and theta activity were observed during neutral playing when compared to distressed (delta: 1.07 (0.25) vs. 3.55 (2.52), theta: 1.08 (0.15) vs. 2.54 (1.79)) and excited (delta: 1.06 (0.30) vs. 3.61 (1.99), theta: 1.08 (0.15) vs. 2.89 (1.49)) conditions. Similarly, frontal/parieto-occipital beta and gamma were reduced in neutral condition when compared to excited state (beta: 1.14 (0.11) vs. 2.54 (1.79), gamma: 1.15 (0.11) vs. 2.95 (1.96)), and parietal-occipital gamma was diminished when neutral condition was compared to distressed playing (gamma: 1.16 (0.15) vs. 2.78 (2.53)). Increased left frontal and parieto-occipital delta and alpha activity were observed in distressed as compared to depressed condition (delta: 2.84 (1.67) vs. 1.30 (0.46), alpha: 1.49 (0.57) vs. 0.92 (0.35)), whereas higher right frontal and parieto-occipital delta and alpha were seen in excited as compared to relaxed condition (delta: 3.56 (2.19) vs. 1.37 (0.63), alpha: 1.83 (0.99) vs. 1.03 (0.29)). Stronger right parieto-occipital delta/theta, and frontal alpha were observed in distressed playing when contrasting to relaxed condition (delta: 3.31 (2.37) vs. 1.32 (0.54), theta: 2.54 (1.79) vs. 1.19 (0.45), alpha: 1.44 (0.50) vs. 0.94 (0.17)). In excited condition, left parieto-occipital theta and alpha were noted when compared to relaxed (theta: 2.89 (1.49) vs. 1.23 (0.58), alpha: 2.31 (1.68) vs. 1.12 (0.44)) and depressed playing (theta: 2.89 (1.49) vs. 1.29 (0.56), alpha: 0.92 (0.35) vs. 1.49 (0.57)). Additionally, excited condition was characterized by higher right frontal alpha and frontal beta when compared to relaxed playing (beta: 2.24 (1.47) vs. 1.18 (0.33)). Elevated frontal gamma was detected during excited playing as compared to depressed (gamma: 2.95 (1.96) vs. 1.22 (0.32)) and relaxed playing (gamma: 3.09 (2.20) vs. 1.51 (0.84)).

**Figure 5:**
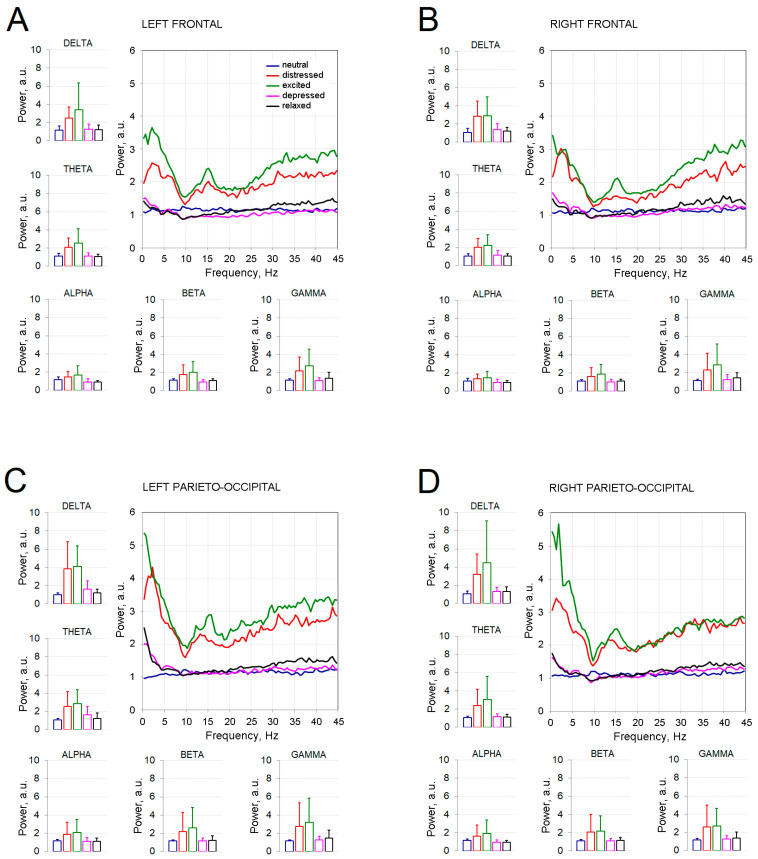
Plots of relative power spectrums obtained for (**A**) left frontal (FP1, AF3, F3), (**B**) right frontal (FP2, AF4, F4), (**C**) left parieto-occipital (P3, PO3, O1) and (**D**) right parieto-occipital (P4, PO4, O2) regions, and corresponding means and standard deviations for each frequency band (delta (1–4 Hz), theta (4–8 Hz), alpha (8–12 Hz), beta (12–30 Hz), and gamma (30–45 Hz). Each color line corresponds to the emotional condition (neutral, distressed, excited, depressed and relaxed). Columns denote means and error bars denote standard deviations.

As the clusters described above contain electrodes from left/right sides and different lobes, to ease the visualization, the spectrum of EEG power relative to baseline neutral playing from the left and right frontal (AF3, F3, Fp1 and AF4, F4, Fp2) and parieto-occipital locations (O1, P3, PO3 and O2, P4, PO4) and means and standard deviations of power per each frequency band for all five emotional conditions is presented in Figure 5.

## 4. Discussion

In this study, we investigated spectral properties of EEG activity in musicians while they were instructed to communicate a certain emotion through performance of a predefined simple music score. The experimental contrast of emotional playing vs. neutral playing was employed to probe brain activity patterns differentially involved in distinct emotional states. In the emotional playing task, participants were instructed to perform in a manner that the targeted emotion is communicated. In contrast, in the neutral playing task, participants were asked to play the same piece neutrally to obtain data for control over general patterns of motor and sensory activation during playing. The tasks of emotional and neutral playing differed considerably with respect to the state of intended-to-transfer emotion arousal and valence levels. The EEG activity differences were observed between distressed/excited and neutral/depressed/relaxed playing.

Similar to other studies on emotions in music [32,33,34,35], we evaluated affective responses within a two-dimensional framework of arousal and valence. However, we instructed our participants to rate how they felt their performance reflected the intended target emotion rather than their actual felt emotions [12,13,14]. This instruction was given in order to take into account that in embodied music performance contexts, musicians emote according to or in response to the intentions of the content they are performing, which does not necessarily reflect their actual felt emotions. To our knowledge, the dichotomy between a person’s actual emotional state and their intended emotional communication through an artistic medium has not been investigated using the EEG signal. Although we cannot contrast experienced emotions versus intended transmission of emotions, in accordance to earlier work, both the neural playing and emotional playing conditions were rated mostly within the expected ranges. Interestingly, the neutral playing was mostly rated as “neutral” (scores around 5) in respect to valence dimension. However, scores on arousal dimension spanned wide range from low to medium values, suggesting that when self-evaluating performances of written music reproduced without expression over many repetitions, arousal levels may fluctuate towards the lower range. This may be an effect of boredom due to repetition.

When EEG activity during neutral playing was contrasted with emotional playing conditions, significant differences occurred in comparison to distressed and excited states. Similarly, these two states showed significant differences from relaxed and depressed conditions. Overall, observed local cortical activations in our study appeared mostly in the frontal and parieto-occipital regions, corresponding to other works addressing emotional processing [36] and supporting earlier findings by Persson (2001) [37] and Lindström (2003) [38], suggesting that a performer should experience certain emotions in process of realizing expressive performance.

Both excited and distressed conditions are characterized by high arousal levels, but differ in the polarity of valence. On the EEG level, significantly higher parietal delta/theta activity was observed in these high arousal states. Previously, Lin et al. (2010) [35] reported emotional arousal to be accompanied by increases in both the delta and theta power spectra when listening to music. Additionally, in the excited condition, elevated beta and gamma power over the parieto-occipital and frontal areas was detected. This might indicate an arousal-related effect as association between beta power elevation following an unspecific increase in emotional arousal was found [39,40]. We cannot not fully rule out the possibility that the elevated gamma activity as observed in the excited condition in comparison to relaxed and depressed conditions is solely of brain origin, since body movement is necessary to play the piano. However, several earlier associations observed between gamma activity and emotional ratings suggest that the detected effect is not entirely due to the muscle activity. Namely, Mao and Run (2014) [41] found gamma power over parietal regions to positively correlate with arousal when subjects listen to the musical intercepts, and Yang et al. (2020) [36] showed an association between gamma activity and experienced emotional arousal. Evenmore, Hadjidimitriou and Hadjileontiadis (2012) and Adamos et al. (2016) [42,43] reported interaction between beta and gamma activity while listening and rating musical preferences, and Bhattacharya and Petsche (2005) [44] reported occurrence of delta and gamma synchronization when musicians listen to musical pieces. This could explain why some effects in low frequency activity parallel those in high frequency range. Further study is needed, employing approaches for finer source-localisation of the EEG signal.

Alternatively, centrally distributed beta activity is known to be related to motor function and originates in the motor areas [45]. Beta oscillations were shown to play an important role in predictive top–down processing along the auditory–motor axis [46,47]. In a study by Schalles and Pineda (2015) [48], beta band power over sensorimotor scalp showed increased suppression during listening to the learned song as contrasted to the scrambled song. Likewise, Aragão Leite et al. (2020) [49] observed an increase in the beta wave activity in the bilateral visual cortexes during complex music execution. Thus, our results of increased beta/gamma in excited playing condition might also reflect more motor effort and increased information transfer during the processing of emotions [50] as required to transfer excited emotion in comparison to neutral, relaxed, and to some extent depressed conditions.

An asymmetric pattern of differences in frontal activation was observed for delta and alpha bands when comparing distressed and excited conditions to depressed and relaxed playing. Specifically, increased left frontal delta and alpha activity emerged in distressed as compared to depressed conditions, while higher right frontal delta and alpha were obtained in excited as compared to relaxed conditions. Dikaya and Skirtach (2015) [18] tasked improvisors to express negative and positive emotions using provided major or minor chord progressions and reported higher theta and beta activation within the frontal left region during the expression of positive emotions, and higher theta and alpha activation in the right hemisphere during the expression of negative emotions. The additional distinction between high and low arousal states in our study may account for these different findings. Indeed, Lee et al. (2020) [51] have previously shown an asymmetric pattern of delta and alpha dynamics when subjects were listening to natural emotional sounds; a left frontal dominance for the relationships between delta and alpha frequency bands was observed in those subjects who experienced positive valence and low arousal levels. The positive valence/low arousal state corresponds to the relaxed condition in the current study that was characterized by somewhat decreased right frontal delta and alpha activity, thus being in line with Lee et al. (2020).

Moreover, the asymmetric hemisphere activation might be related to motivational direction (i.e., arousal aspect), rather than affective valence [52]. Alpha activity is commonly associated with general arousal and various inhibitory processes in the brain. In line with this notion, Rogenmoser et al. (2016) [53] reported association of power suppression in parietal alpha frequency band with higher arousal while listening to musical pieces. In contrast, we observed higher parietal alpha in excited as compare to both depressed and relaxed conditions that are defined by high arousal. However, this discrepancy could stem from the fact that our subjects were performing and not freely listening to the music, and hence alpha change may not entirely reflect arousal level. Indeed, alpha activity originating from parieto-occipital regions was previously related to internally-oriented attention states such as imagery [54,55] which may describe a process of immersion into the music as proposed by Jäncke et al. (2015) [56]. Furthermore, increased alpha synchronization in parieto-temporal brain regions was associated with brain activation during creative processing [57].

There are a limited number of studies assessing EEG dynamics while subjects are actively performing a piece. Expression of emotion through music improvisation is typically achieved through deviating from expectancy in a creatively nuanced manner through establishing a theme then variating upon it [20]. Subjects in our study were provided the established theme upon which to variate. Previously, it was shown that the pattern of activation might depend on the improviser’s experience/performance quality; high-quality jazz performances were associated with right frontal clusters of theta, alpha, and high-beta activity in Rosen et al. (2020) [20] and predominant activation in the left hemisphere, and simultaneous inter-hemispheric integration in the beta frequency band between the frontal right and parietal left regions in professional musicians but not in amateur musicians [18] was observed. As suggested by Rosen et al. (2020) [20], the activation pattern depends on whether creativity is defined in terms of the quality of products or the type of cognitive processes involved. In addition, a recent study by Sasaki et al. (2019) [19] in active guitar players contrasted improvisation and scale playing and revealed greater activity for improvisation over scale in multiple frequency bands (theta, alpha, and beta) localized in the frontal, temporal, motor and parietal areas. The authors suggest that improvisation is mediated by processes involved in the coordination of planned movement sequences that are modulated in response to an ongoing environmental context through monitoring and feedback of sensory states in relation to internal plans and goals. Thus, a subject’s level of experience is very likely to introduce variability and contribute to the discrepancies in the findings.

The major limitation of our study is the small sample size. However, each subject was enrolled four times under well-controlled experimental settings, followed by a clear pipeline of data evaluation. Our results together with previous reports suggest that expressive playing, requiring certain amount of improvisation, does not only involve emotional aspects, but also motor and cognitive aspects that need to be taken into account when evaluating EEG data during embodied music interaction contexts such as active playing and improvisation.

## Data Availability

The data presented in this study are available on request from the corresponding author. The data are not publicly available due to privacy restrictions.

## Supplementary Materials

The following are available online at https://www.mdpi.com/article/10.3390/s21227466/s1, Table S1: The detailed description of the instruction procedure.
