# Supplementary material for: Spectral Characteristics of EEG during Active Emotional Musical Performance
Source: Sensors (Basel). 2021 Nov 10;21(22):7466. doi: 10.3390/s21227466 (PMC8620396; doi:10.3390/s21227466)
Supplement: Supplementary file 1 [file sensors-21-07466-s001.zip › sensors-1413366-supplementary.pdf]

Supplementary material: Slides used to brief participants regarding the experiment protocol.

Table S1. The detailed description of the instruction procedure.

**Brain Recording Sessions**<br>JVLMA Music Psychology Lab	The purpose of this briefing is to inform you regarding what to expect when you come for your first recording session, and to answer any questions you may have.
**Main Question**<br>What is happening in musician's brains when they are playing expressively?<br>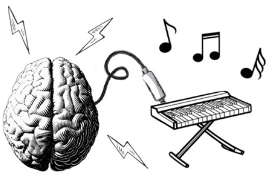	To understand what to expect, you should know the goal of the recording sessions.<br><br>This study investigates what the brain is doing when musicians are expressing emotions while playing music.
**Main Task**<br>Repeat the same 1 minute piece many times.<br>Change the intended emotion each time.<br>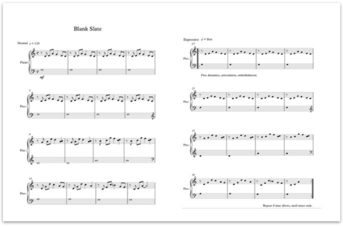 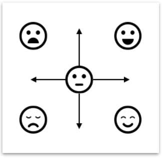	The task will be to play the same 1 minute piece many many times - while only changing the intended emotion to be expressed. Here on the left is the written score, where the first page is to be played strictly as written, and the second page is to be played expressively. Here on the right the emotions are shown as emojis plotted on a 2 dimensional affective space for illustrative purposes. A more detailed explanation of each of these and the 2D affective space will follow.

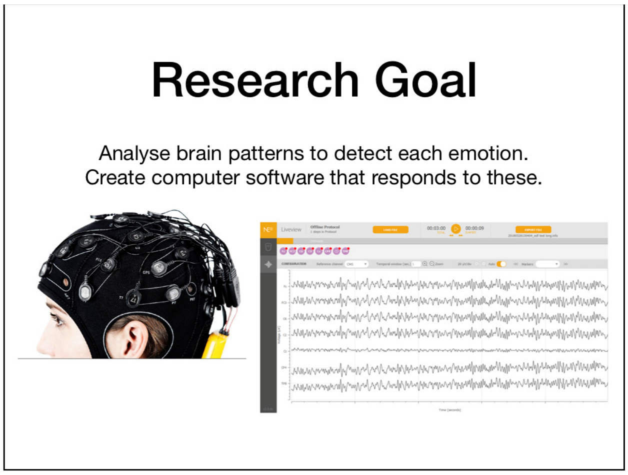

After recording, a team of neuroscientists will analyse your EEG data to find patterns that may correspond to each emotion.

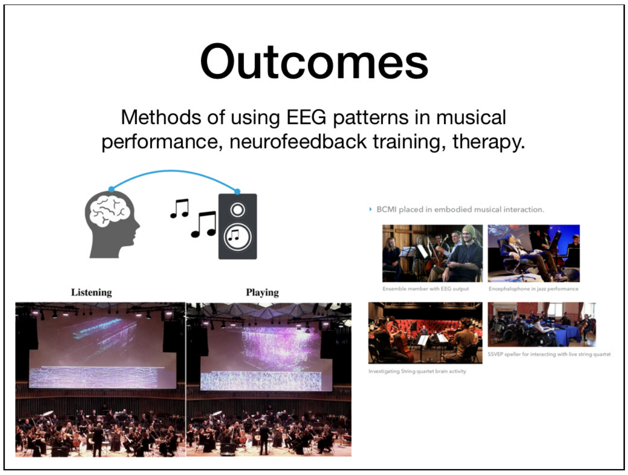

These patterns will give us clues of how to use the EEG to support or enhance musical interaction, performance and training in the future. We intend to present our findings of your brain recordings at the end of 2021.

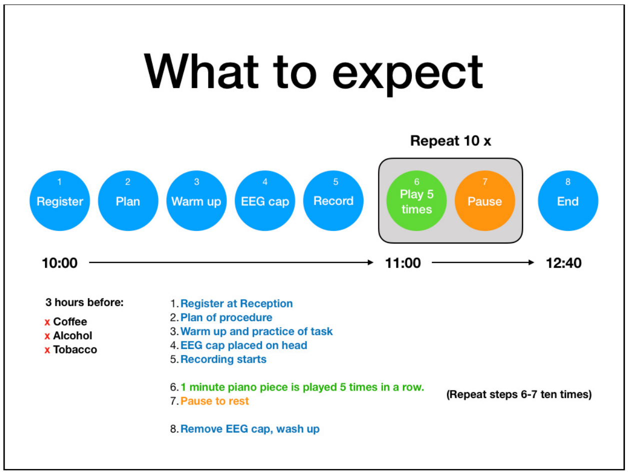

This is what to expect at each recording session. It is important that we agree not to consume any coffee, alcohol or tobacco products at least 3 hours before your session that day.

The session will consist of:

1. Explanation of procedure
2. Warm up and practice of task
3. EEG cap placed on head
4. Recording starts
5. 1 minute piano piece is played 5 times in a row.
6. Break
7. Repeat steps 5-6 ten times
8. Recording ends
9. Remove EEG cap, wash up

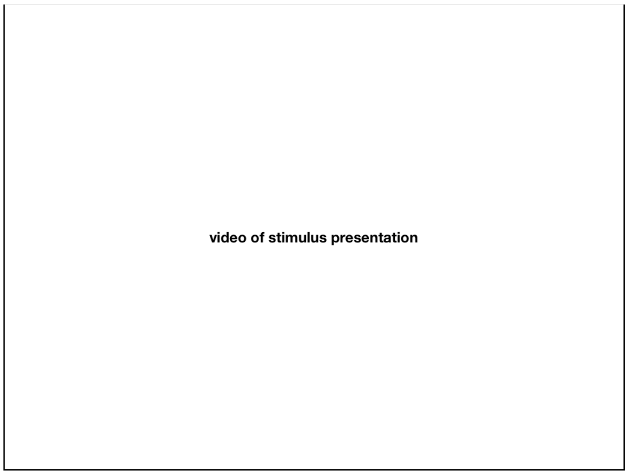 	For step 5, here is a video of the instructions you will see during the actual recording. It will be shown on a laptop at eye level on a piano.
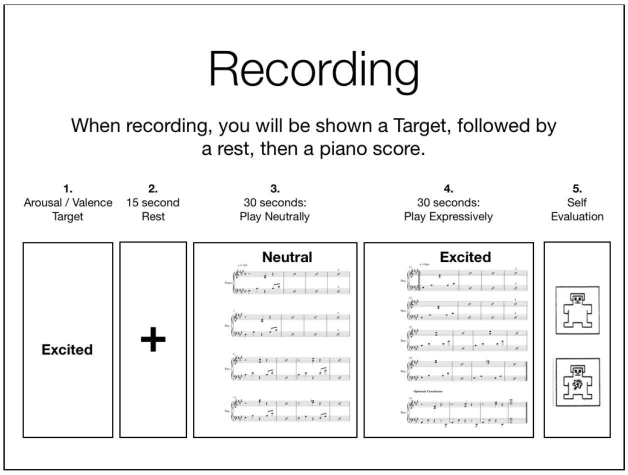 	Here shows a summary of the recording protocol for one trial in order, using the example of the target emotion “Excited”.<br><br>You can see that the order of each trial will include:<br><br>1. You will be shown the target emotion for this trial<br>2. Rest for 15 seconds, be calm and look at the cross onscreen. This step is to record a baseline resting state.<br>3. Play the first page of the score as written (without emotion) for 30 seconds.<br>4. Play the second page of the score expressively, with intent to communicate the target emotion through your playing to a listener.<br>5. Evaluate your own performance.

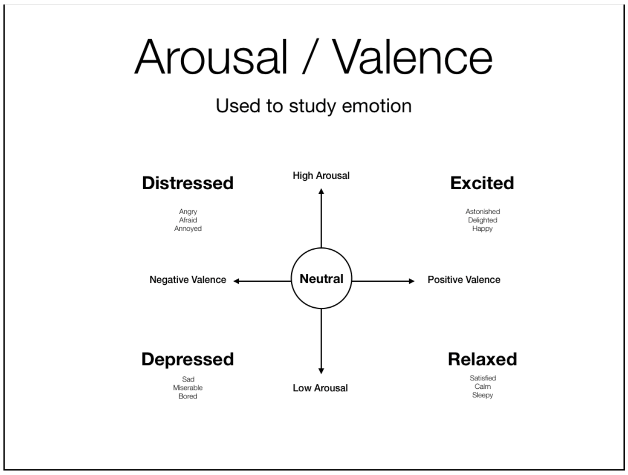

Arousal and Valence are dimensions by which psychologists model, measure and study the affective space- our moods and emotions.

The words chosen to describe the emotions in this model for this study are “Distressed”, “Excited”, “Depressed”, “Relaxed” and “Neutral”.

There are other words used in other research studies to describe the same quadrants in this model (some examples shown here), but the important thing to understand is that Valence refers to the negative to positive dimension, while Arousal refers to the dimension of physiological alertness.

So in this case, we consider Distressed and Depressed to be emotions on the negative/low valence spectrum, and Excited and Relaxed to be emotions on the positive/high valence spectrum. In the same way, Distressed and Excited are considered high arousal emotions, whereas Depressed and Relaxed are low arousal emotions.

Neutral here is represented in the middle of both scales. Whenever performing the score as written, one should aim for Neutral in this model.

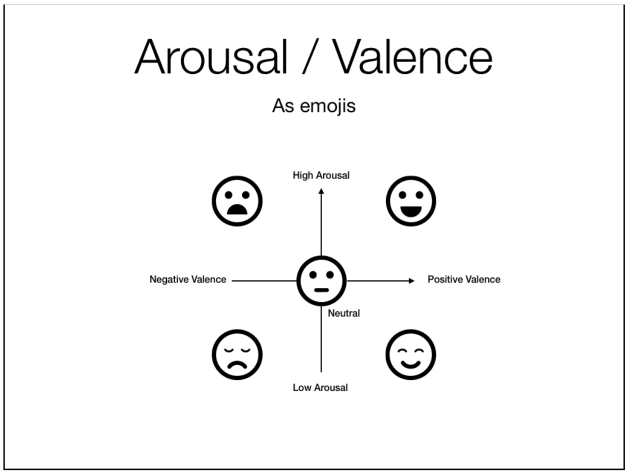

Once again, just for illustration purposes, here is how the 2D model of affect may be represented as emojis. To standardise our methods with the surrounding research community we choose to use words in English rather than emojis, but these can help illustrate the idea for better understanding.

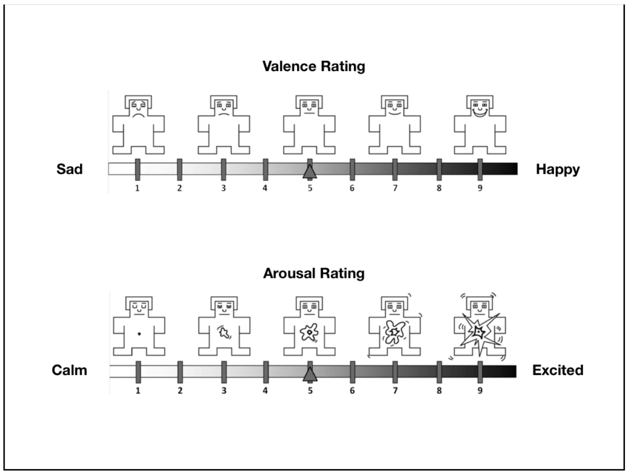

These dimensions of Arousal and Valence are important to understand because after each play through of the score, you will be asked to self-evaluate your performance on these scales. It is not meant to be a rating of technical aspects of your performance, but rather a way to track how on target you perceive you are. To be clear:

A high score for Depressed would be:
Arousal 1
Valence 1

A high score for Distressed would be
Arousal 9
Valence 1

A high score for Relaxed would be
Arousal 1
Valence 9

A high score for Excited would be
Arousal 9
Valence 9

For example, if the emotion targeted was Distressed, but you felt that you didn't capture that emotion very strongly in this particular performance, you could indicate:
Arousal 6
Valence 3

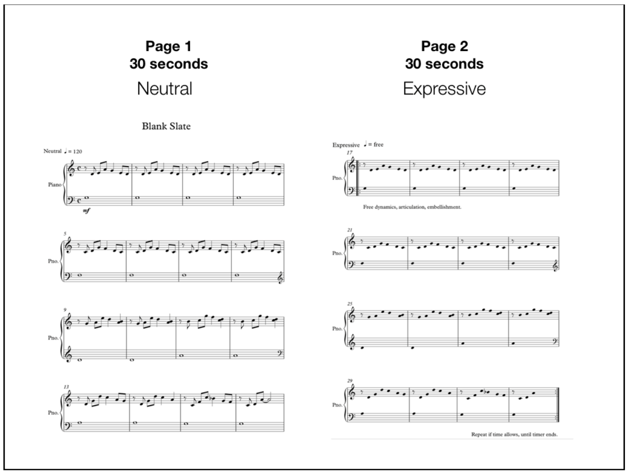

The music score is designed to be simple enough for pianists of your experience to be able to pick up quickly and make variations upon. It is written in an expanded pentatonic scale to avoid the typical western cadence. Note that while the second page is a repeat of the pitch content of the first page, rhythm and tempo have been removed, and freedom is allowed for dynamics, articulation, embellishment and any other parameters at your disposal in your effort to communicate the target emotion. You are invited to create a variation or an improvisation on the established material in the expressive playing segment.
